# How digital leadership impacts community resilience: a moderated mediation model

**DOI:** 10.3389/fpubh.2025.1524985

**Published:** 2025-06-16

**Authors:** Guanhu Zhao, Fazhen Zhao, Xu Hui, Yanan Wu, Xuemei Zhao

**Affiliations:** ^1^School of Management, Lanzhou University, Lanzhou, China; ^2^Research Center for Emergency Management, Lanzhou University, Lanzhou, China; ^3^Lanzhou University Library, Lanzhou, China; ^4^Evidence-Based Medicine Center, School of Basic Medical Sciences, Lanzhou University, Lanzhou, China; ^5^Centre for Evidence-Based Social Science/Center for Health Technology Assessment, School of Public Health, Lanzhou University, Lanzhou, China; ^6^School of Psychology, Guizhou Normal University, Guiyang, China

**Keywords:** digital leadership, community resilience, knowledge sharing, community trust, moderated mediation model

## Abstract

**Introduction:**

Community resilience is crucial for communities to effectively respond to disasters such as public health emergencies. Digital technology and leadership are integral to building community resilience; however, the impact of digital leadership on community resilience has been underexplored.

**Methods:**

This study administered a questionnaire survey to 306 participants to examine the impact of digital leadership on community resilience. Furthermore, it also explored the mediating role of knowledge sharing and the moderating role of community trust.

**Results:**

We find that (1) digital leadership, knowledge sharing, community trust and community resilience are positively correlated with each other; (2) knowledge sharing partially mediates the relationship between digital leadership and community resilience; and (3) community trust moderates the effect of digital leadership on knowledge sharing. Specifically, under the condition of high community trust, digital leadership is more effective in predicting community resilience.

**Discussion:**

The findings of this study not only contribute to the existing literature on the antecedents of community resilience but also elucidate the influence mechanism of digital leadership on community resilience from a micro perspective. Furthermore, this study provides practical recommendations for enhancing community resilience in the digital era.

## 1 Introduction

As of 13 October 2024, the COVID-19 public health outbreak had caused 776,618,091 infections and 7,071,324 deaths worldwide (1). Unpredictable shocks, including natural and man-made disasters, have increasingly caused severe impacts on communities (2–4). The UN World Conference on Disaster Risk Reduction recommended designating communities as the fundamental units for disaster risk reduction, emphasizing the priority of building community resilience. During the pandemic, hundreds of millions of people stayed at home to work, with 50% of Americans and 38% of Britons engaging in remote work (5, 6). Social distance and lockdown regulations require individuals to avoid physical contact with others (7). The rapid adoption of digital technologies during the COVID-19 pandemic was driven by the need for social distancing and remote working arrangements (8). Digital technologies allow organizational members to stay connected while maintaining social distance (9). For instance, the utilization of social media enables community members to engage in frequent and meaningful communication, which assists in addressing emotional concerns and daily life challenges, thereby enhancing their overall quality of life (10, 11). Researchers have highlighted the role of digital technologies in addressing uncertainty and enhancing community resilience (12, 13). Given that digital leadership facilitates the integration of new digital technologies into the workplace (14), the impact of digital leadership on community resilience has emerged as a critical research topic.

Community resilience refers to the capacity of communities to utilize their resources to prepare for, respond to, endure, and recover from extreme events like disease outbreaks (15). Leadership has garnered significant attention from scholars in the study of community resilience (16, 17). Studies have demonstrated that strengthening the leadership in a community is the starting mechanism for activating community resilience (18). Meanwhile, a substantial body of literature highlights the positive impact of leadership on community resilience (19–21). Digital leadership is a new concept in which a leader's management functions are enabled by digital technologies and digital platforms (22). Digital leadership can help organizations deal with risk and ongoing uncertainty (23). In the context of challenges posed by emerging technologies like Artificial Intelligence and the Internet of Things (IoT), digital leadership has emerged as a key factor in enhancing community resilience (12, 24, 25). Additionally, prior research has highlighted the beneficial effects of digital leadership on various dimensions, including innovation performance (26), teamwork role performance (27), employee creativity (14) and safety performance (28).

Although digital leadership has been extensively examined in the private sector, empirical studies on its application within community contexts remain relatively scarce (29). In the field of community resilience research, there is a notable lack of quantitative studies that explore the impact of digital leadership. Significant gaps exist in our understanding of the specific contexts and mechanisms through which digital leadership influences community resilience. Looking at previous literature indicates that existing research has paid insufficient attention to knowledge sharing when examining the relationship between digital leadership and community resilience. Knowledge sharing can enhance the collaborative capacity of community organizations, which is crucial for improving community resilience (30). Moreover, leadership performance is invariably shaped by contextual factors that warrant further exploration. Much of the existing studies on digital leadership overlook the role of trust. Community trust, as an important contextual factor (31), significantly influences community resilience (32) and may mediate the relationship between digital leadership, knowledge sharing, and community resilience. This study attempts to explore and answer the following four research questions:

RQ1. Does digital leadership relate to community resilience?
RQ2. Does digital leadership relate to community resilience through the mediating effects of knowledge sharing?
RQ3. Does community trust moderate the relationship between digital leadership and knowledge sharing?
RQ4. Does community trust moderate the mediating pathway?

The moderated mediation model helps to elucidate the underlying mechanisms and boundary conditions of a relationship (33–35). To answer these questions, this study proposes a moderated mediation model that delivers substantial theoretical and practical contributions. First, this study tried to extend the antecedents of community resilience by adding digital leadership. Existing literature has shown that leadership is one of the critical aspects influencing community resilience (17, 36, 37). Our research is one of the first studies to bridge the link between digital leadership and community resilience. Second, we seek to clarify how digital leadership affects community resilience by investigating the mediating role of knowledge sharing. Third, through the moderating role of community trust, we identify the specific conditions under which digital leadership influences community resilience through knowledge sharing. Our findings provide valuable insights that may contribute to managerial implications for enhancing community resilience in the digital era.

Following the introduction, the structure of the article is organized as follows. Firstly, this study introduces the theoretical basis and relevant literature to develop research hypotheses and conceptual framework. Secondly, the research methods of the paper are presented. Next, our findings are reported, followed by a discussion of the results. Finally, the theoretical contributions and managerial implications are stated, together with the limitations and directions for future research.

## 2 Theoretical basis and research hypotheses

### 2.1 Theoretical basis

#### 2.1.1 Social exchange theory

Among the theories related to knowledge sharing, social exchange theory is one of the most widely applied theories (38). Homans (39) initially introduced the concept of social exchange theory, positing that the exchange of information between individuals and between individuals and organizations constitutes a social exchange. Knowledge sharing is the exchange of task-related information, advice, and expertise to help others and to collaborate with others to solve problems (40, 41). With the development of online communication platforms, digital leaders can leverage information technology to effectively enhance communication among community members. This not only promotes knowledge sharing but also enhances their participation in disaster reduction actions, ultimately strengthening community resilience. Therefore, based on the social exchange theory, this paper examines the mediating role of knowledge sharing in the relationship between digital leadership and community resilience.

#### 2.1.2 Motivated information processing in groups theory

Motivated information processing in groups (MIP-G) theory suggests that individuals are driven by a combination of prosocial and pro-self motives, with prosocial motives directing attention toward collective outcomes (42). Higher levels of trust enhance members' willingness to achieve organizational goals (43). Community trust refers to the trustworthiness of those in our immediate physical surroundings—that is, fellow residents in the neighborhoods, in communities, and in municipalities (44, 45). Consequently, when community trust is high, emotional bonds may redirect community members' focus from personal interests to collective wellbeing. This shift increases residents' motivation to share knowledge about disaster response with their neighbors. Drawing on the MIP-G theory, this study proposes that community trust moderates the relationship between digital leadership and knowledge sharing.

### 2.2 Research hypotheses

#### 2.2.1 Digital leadership and community resilience

Digital technologies can mitigate social challenges and enhance resilience during the COVID-19 pandemic (46). Leaders with digital leadership are providing their respective organizations with digital expertise and technological infrastructure to enhance resilience in crisis-induced environments (47). The extant literature indicates that digital leadership considerably affects organizations' capacity to achieve sustainable performance (48, 49). Consequently, those who demonstrate digital leadership are more likely to guide their organizations in fostering adaptive capacity in the context of evolving circumstances, thereby ultimately enhancing sustainability (49). Specifically, digital leadership has the potential to improve the velocity of information dissemination within a community markedly. This can facilitate prompt access to the most recent guidance for community members while also bolstering the capacity of residents to effectively respond to emergencies (50).

In addition, digital leadership can facilitate collaboration among community members. Leaders can use digital technologies to create collaborative platforms that facilitate the exchange of resources and experiences among community members. Communities with strong collaborative capacities have been observed to show greater resilience during adversity (51, 52).

Therefore, we posit that digital leadership facilitates community resilience and suggest the following hypothesis:

H1: Digital leadership can significantly improve community resilience.

#### 2.2.2 The mediating role of knowledge sharing

Leadership is a crucial driver of the knowledge management process in community organizations. Digital leadership represents an emerging paradigm in leadership that leverages digital technologies to facilitate knowledge sharing. Digital leadership means that leaders combine digital capabilities with leadership skills (53, 54). Digital leadership is well-positioned to spearhead and advance the practice of disaster knowledge management within a community, drawing upon its comprehensive grasp of technology. For example, digital leadership can facilitate the rapid dissemination of knowledge through social media platforms. As demonstrated, digital leadership exerts a positive and significant influence on knowledge sharing (22).

Effective disaster response depends not only on governmental actions but also on the knowledge and actions possessed by community residents (55, 56). The case study findings indicate that knowledge is a significant factor in determining resilience (57). Specifically, community resilience is significantly contingent upon the ability to foster knowledge sharing among key stakeholders (58, 59). Additionally, research demonstrates that knowledge sharing supports community organizations in post-disaster healthcare activities, further enhancing community resilience (60). Thus, knowledge sharing is a vital component in promoting community resilience.

As previously discussed, digital leadership may positively influence both knowledge sharing and community resilience. Meanwhile, knowledge sharing is likely to have a positive impact on community resilience. Furthermore, knowledge sharing is a pro-social behavior (61). Based on the social exchange theory, knowledge sharing may be a mediator between digital leadership and community resilience. Based on this reasoning, we propose the following hypothesis:

H2: Knowledge sharing mediates the relationship between digital leadership and community resilience.

#### 2.2.3 The moderating role of community trust

Drawing on the MIP-G theory, this study proposes that community trust serves as a moderator in the relationship between digital leadership and knowledge sharing. Similarly, trust moderates the relationship between servant leadership and knowledge-sharing tendency (62). The level of trust between organizational members can affect the influence of digital leadership and knowledge sharing (63). If community members lack trust in each other or their leaders, they may be less inclined to engage in knowledge share (64). Insufficient community trust can weaken the positive association between digital leadership and knowledge sharing. That is, when community trust is at a low level, the impact of digital leadership on knowledge sharing among community residents is diminished. Conversely, when community trust is at a high level, digital leadership can more effectively coordinate knowledge sharing among community residents. Drawing from this argument, we suggest the following hypothesis:

H3: Community trust moderates the relationship between digital leadership and knowledge sharing.

In the digital era, leadership prioritizes fostering trust (65). Studies have shown that managers should promote knowledge sharing within the workplace by building trust in social interactions (66). Community trust can either strengthen or weaken the relationship between digital leadership and community resilience through knowledge sharing. Specifically, when community trust is high, the positive impact of digital leadership on community resilience through knowledge sharing is enhanced; conversely, when community trust is low, this relationship is diminished. Therefore, community trust may moderate the pathways through which digital leadership influences community resilience through knowledge sharing. Consequently, we propose the following moderated mediation hypotheses for further investigation:

H4: Community trust moderates the mediating relationship between digital leadership and community resilience through knowledge sharing.

**Figure 1:**
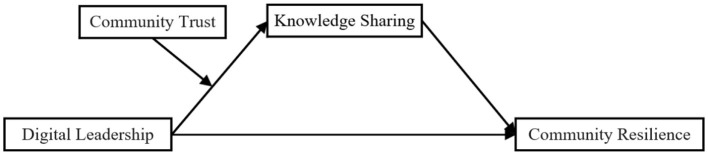
Conceptual framework.

Figure 1 represents this study's empirical model.

## 3 Research methods

### 3.1 Study design

#### 3.1.1 Participants

In China, urban communities serve as the fundamental units of urban governance, constituting the lowest administrative level of government (67, 68). During the COVID-19 pandemic, approximately 4 million community workers were engaged in community outbreak prevention and control efforts (69). This study employs a cross-sectional survey design, targeting urban community workers in Gansu and Guizhou provinces. Participants were recruited via a snowball sampling method.

Inclusion criteria: (1) A minimum of 1 year of experience working in the community; (2) Participation in community emergency management activities; (3) Voluntary participation with informed consent. Exclusion Criteria: Participants who did not have a smartphone or were unable to use a computer to complete the survey will be excluded from the online surveys.

#### 3.1.2 Procedure

This study conducted both online and offline formal surveys. The offline survey was conducted by distributing questionnaires within the community. Initially, the researchers independently distributed a limited number of questionnaires. Subsequently, additional participants were selected through referrals from respondents who had already completed the questionnaires. The online survey was conducted via the QuestionStar platform. Participants were able to forward the questionnaire link to friends in their WeChat contacts whom they deemed suitable for participation.

All questionnaires were distributed during the same period. The snowball sampling process continues until an adequate sample size is achieved. Participants first provided their demographic information, followed by completing the digital leadership scale, knowledge sharing scale, and community trust scale. Finally, community resilience was measured.

#### 3.1.3 Quality control

Questionnaires exhibiting inconsistent responses (e.g., identical answers across all items, suggesting a lack of diligence in completing the questionnaire) were manually excluded during the data preprocessing phase. To prevent duplicate submissions in the online survey, each IP address was restricted to a single use.

#### 3.1.4 Ethical considerations

This study was approved by the Academic Committee of the Non-clinical School of Management, Lanzhou University. Before participating in the questionnaires, all participants provided informed consent. All information collected was kept confidential and anonymous.

#### 3.1.5 Sample size calculation

Given that the PROCESS macro employs a multiple regression framework, a linear multiple regression model was utilized for statistical analysis. The minimum sample size was calculated using the G^*^Power 3.1.9.7 software with parameters set at a significance level (α) of 0.05, a medium effect size *f*^2^ of 0.15, statistical power (1 – β) of 0.8, and four predictors in the multiple linear regression model. The required minimum sample size was calculated to be 85 participants. Power analyses conducted using G^*^Power have consistently demonstrated that a sample size of *N* = 85 is adequate for the most complex analyses (70–72). In this study, 350 questionnaires were distributed, of which 306 were valid, resulting in a validity rate of 87.43%. Therefore, our effective sample size exceeding 85 is reasonable and meets the necessary criteria.

This study involved 306 participants, comprising 155 males (50.7%) and 151 females (49.3%). The majority of the participants, 68.3%, were aged between 20 and 50 years. Furthermore, 83.9% of the participants had received higher education, with over half holding an associate's degree or higher. Table 1 provides detailed demographic information of the survey participants.

**Table 1:** Participants ' demographic information (*N* = 306).

**Variables**	**Classification**	**Number**	**Percentage (%)**
Gender	Male	155	50.7
	Female	151	49.3
Age	≤ 20	82	26.8
	20–30	124	40.5
	30–40	59	19.3
	40–50	26	8.5
	≥50	15	4.9
Education level	Junior high school	10	3.3
	High school	39	12.7
	Associate's degree	195	63.7
	Bachelor's degrees	53	17.3
	Master's degrees	9	2.9

### 3.2 Measurements

All scales underwent translation and back-translation to verify content accuracy. Some items were revised to more accurately reflect the relevant characteristics of the community. A 7-point scale permits more significant variability in the data than a 5-point scale (73). Furthermore, a broader range of scores around the mean facilitates more comprehensive conceptual recognition and a more accurate and effective capture of respondents' attitudes. We adjusted the original four- or five-point scales to a seven-point Likert scale, where 1 represents “strongly disagree” and 7 represents “strongly agree”.

According to existing studies, we utilized the CCRAM-10 to measure community resilience (74). CCRAM-10 comprises 10 items, which is a comprehensive indicator indicating a stronger perceived sense of community resilience. Examples include “There is mutual assistance and people care for one another” and “I count on my community to assist and share essential information” (75, 76).

Digital leadership is adapted from Zeike et al. (77), which contains six items. For example, “I think using digital tools is fun.” and “I am driving the digital transformation forward proactively in our unit.”

Knowledge sharing is adapted from Bock et al. (78), which contains four items. For example, “I share my experience or know-how from work with other community members more frequently in the future.” and “I will always provide my know-where or know-whom at the request of other community members.”

Community trust is adapted from Wollebaek et al. (79), which contains three items. For example, “I trust the people living in my neighborhood.” and “I trust my neighbors.”

### 3.3 Data analysis

First, this study employed the widely recognized Harman single-factor test method to test the common method bias in this study (80). Second, to evaluate the reliability and validity, this study has reported Cronbach's alpha (CA), composite reliability (CR), and average variance extracted (AVE). These are most widely used in existing literature to evaluate reliability and validity (81, 82). Third, this study adopted the approach proposed by Henseler et al. (83), employing the measurement invariance of composite models (MICOM) and non-parametric partial least square multi-group analysis (PLS-MGA) to assess the measurement invariance (84). Finally, the mediation model was tested using PROCESS Macro Model 4, and the moderated mediation model was examined using PROCESS Macro Model 7.

## 4 Results

### 4.1 Common method bias test

Common method bias, a systematic error, can severely distort research findings and lead to misleading conclusions (85). Therefore, this paper utilized the Harman single-factor method to assess common method bias. The test results indicate that factors with eigenvalues exceeding 1 account for 76.54% of the total explained variance. The first principal component accounts for 32.85% of the variance, which is below the critical threshold of 50% (86). Consequently, this study has no serious common method bias.

### 4.2 Reliability and validity tests

Accurate research results depend on strong construct reliability and validity. The questionnaire's reliability can be reflected by the Cronbach's alpha values of all constructs. The construct reliability is deemed good when the alpha value exceeds 0.70 (87). Table 2 shows that all Cronbach's alpha values range from 0.894 to 0.964, confirming our study's high construct reliability.

**Table 2:** The average variance extracted, composite reliability and Cronbach's α coefficients.

**Latent variable**	**Items**	**Loadings**	**AVE**	**Composite reliability**	**Cronbach's α**
Digital leadership	DL1	0.696	0.7354	0.9429	0.941
	DL2	0.858			
	DL3	0.843			
	DL4	0.837			
	DL5	0.952			
	DL6	0.935			
Knowledge sharing	KS1	0.849	0.7663	0.9291	0.928
	KS2	0.897			
	KS3	0.906			
	KS4	0.848			
Community trust	CT1	0.820	0.7386	0.8944	0.894
	CT2	0.875			
	CT3	0.882			
Community resilience	CR1	0.875	0.7243	0.9632	0.964
	CR2	0.864			
	CR3	0.798			
	CR4	0.790			
	CR5	0.835			
	CR6	0.833			
	CR7	0.822			
	CR8	0.836			
	CR9	0.921			
	CR10	0.925			

The convergent validity of each item was examined. The indicator factor loadings are significant and exceed the acceptable value of 0.6 on their corresponding constructs (88, 89). The factor loadings are significant and surpass the acceptable value of 0.6 for their corresponding constructs. The average variance extracted (AVE) is >0.5, demonstrating good convergent validity (88, 90). The Composite reliability exceeds 0.89, demonstrating acceptable internal consistency reliability (89). As shown in Table 2.

As shown in Table 3, digital leadership, knowledge sharing, community trust and community resilience are positively correlated with each other. Meanwhile, the square root of AVE surpasses the correlation coefficients in the same column of Table 3, demonstrating high discriminant validity between variables (91).

**Table 3:** Correlations, means, standard deviations and the square root of AVE.

**Variables**	**Mean**	**SD**	**1**	**2**	**3**	**4**
DL	4.847	0.881	0.858			
KS	4.909	0.968	0.558^**^	0.875		
CT	4.003	1.005	0.796^**^	0.632^**^	0.859	
CR	4.591	1.101	0.579^**^	0.657^**^	0.476^**^	0.851

N = 306; the italic numbers on the diagonal line represent the square root value of AVE; significance levels are indicated as ^*^p < 0.05, ^**^p < 0.01, ^***^p < 0.001, same below.

### 4.3 Measurement invariance test

This study assesses measurement invariance between the offline and online samples. SmartPLS version 4.1.1.1 was used for the measurement invariance test (92).

MICOM results are displayed in Table 4, where the original correlations are equal to or exceed the 5% quantile. Moreover, the mean original difference values and variance original values lie within their corresponding 95% confidence intervals, confirming that the two groups (offline and online samples) of data achieve full measurement invariance (93).

**Table 4:** Results of invariance measurement testing using permutation.

**Constructs**	**Configurational invariance (step 1)**	**Compositional invariance (step 2)**		**Partial measurement invariance**	**Equal mean assessment (step 3a)**		**Equal variance assessment (Step 3b)**		**Full measurement invariance**
		**Original correlation**	**5.00%**		**Original differences**	**Confidence interval**	**Original differences**	**Confidence interval**	
CR	Yes	1.000	1.000	Yes	−0.021	[−0.220; 0.236]	0.026	[−0.340; 0.346]	Yes/Yes
CT	Yes	0.999	0.999	Yes	−0.109	[−0.209; 0.216]	−0.141	[−0.281; 0.290]	Yes/Yes
DL	Yes	1.000	0.999	Yes	0.003	[−0.213; 0.220]	−0.207	[−0.359; 0.331]	Yes/Yes
KS	Yes	1.000	1.000	Yes	−0.065	[−0.200; 0.245]	−0.17	[−0.486; 0.430]	Yes/Yes

In addition, the study compares the path coefficients between the two groups using the PLS-MGA approach (83). The results in Table 5 indicate that there were no significant differences in any of the path coefficients between the two groups (84). Therefore, the two groups can be pooled for analysis (94).

**Table 5:** Results of multi-group analysis.

**Associations**	**Path coefficients diff. (offline samples–online samples)**	***p-*value (offline samples vs. online samples)**	**Decision**
DL → KS	0.139	0.375	No difference
KS → CR	0.026	0.812	No difference
DL → CR	−0.04	0.717	No difference
DL → KS → CR	0.076	0.372	No difference
CT × DL → KS	−0.012	0.901	No difference
CT × DL → KS → CR	−0.003	0.949	No difference

### 4.4 Hypotheses testing

Table 6 presents the results of the direct effects, mediation analysis, and moderation tests. Hayes (95) Models 4 and 7 of the PROCESS macro were utilized to test the hypothesized model. The number of bootstrapped samples was set to 5,000, and a 95% confidence interval was specified.

**Table 6:** Conditional process analysis.

**Predicators**	**CR**		**KS**		**CR**		**KS**	
	**β**	***t***	**β**	***t***	**β**	***t***	**β**	***t***
Gender	−0.26	−2.49^*^	−0.13	−1.45	−0.18	−2.03^*^	−0.2	−2.35^*^
Age	0.02	0.38	0.03	0.72	0.00	0.03	0.04	0.98
Education level	−0.05	−0.79	−0.08	−1.36	−0.01	−0.15	−0.05	−0.92
DL	0.72	12.37^***^	0.61	11.66^***^	0.39	6.36^***^	0.24	2.81^*^
KS					0.54	9.61^***^		
CT							0.46	6.34^***^
DL x CT							0.12	2.94^**^
*R* ^2^	0.35		0.32		0.50		0.44	
F	40.74^***^		35.90^***^		60.98^***^		39.09^***^	

Significance levels are indicated as ^*^p < 0.05, ^**^p < 0.01, ^***^p < 0.001.

In line with Hypothesis 1, digital leadership had a significant positive effect on community resilience (β = 0.72, *p* < 0.001); thus, the H1 of the study was accepted. Furthermore, digital leadership was significantly associated with knowledge sharing (β = 0.61, *p* < 0.001). When digital leadership and knowledge sharing were included in the regression equation, they had a significantly positive effect on community resilience (Table 6). As presented in Table 7, the bootstrapping results confirmed that the indirect effect of digital leadership on community resilience through knowledge sharing supported mediation as the estimated 95 percent confidence interval [0.23, 0.34] did not contain zero. The mediating effect accounts for 45.83%; thereby H2 was supported. Additionally, since the direct effects of digital leadership on community resilience also did not contain zero, this indicates the presence of a partial mediation model.

**Table 7:** The results of the mediation effect test.

**Effect**	**β**	***SE***	**LLCI**	**ULCI**	**Effect ratio**
Total effect	0.72	0.06	0.61	0.83	100%
Direct effect	0.39	0.06	0.27	0.51	54.17%
Indirect effect	0.33	0.05	0.23	0.34	45.83%

**Figure 2:**
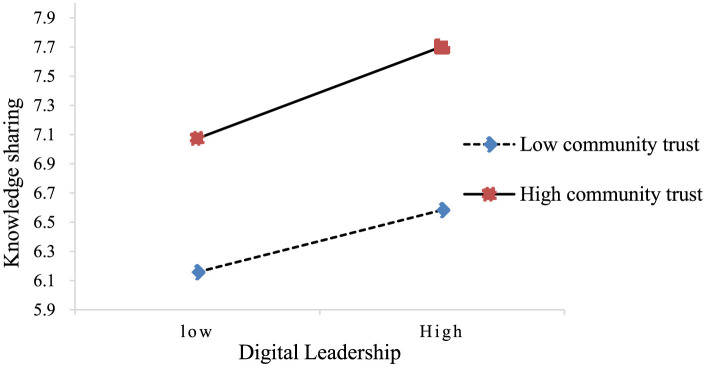
The moderating effect diagram of community trust between digital leadership and knowledge sharing.

Before testing Hypothesis 3, digital leadership and community trust were mean-centered by employing Hayes's (95) Process macro. As presented in Table 6, the interaction term of digital leadership and community trust was a significantly positive predictor of knowledge sharing (β = 0.12, *p* < 0.01), 95% confidence interval was (0.04, 0.19), excluding 0. A simple slope test was conducted by using the values of community trust plus and minus one standard deviation (96) (see Figure 2). The results indicated that digital leadership was a significant positive predictor of knowledge sharing at high levels of community trust (simple slope = 0.36, *t* = 3.33, *p* < 0.001), indicating that higher levels of community trust were associated with stronger effects of digital leadership on knowledge sharing. This result showed that community trust played a moderating role in the relationship between digital leadership and knowledge sharing. Hence, H3 is also supported.

Table 8 presented the results of the conditional indirect effect of digital leadership on community resilience through knowledge sharing at high and low values (±1 SD from mean) of community trust.

**Table 8:** The results of the conditional indirect effect.

**Level and level value**	**Effect**	**Boot SE**	**Boot LLCI**	**Boot ULCI**
CT Low −1SD (−1.01)	0.07	0.05	−0.02	0.17
CT Mean (0)	0.13	0.05	0.03	0.24
CT High +1SD (1.01)	0.19	0.07	0.06	0.34
**Index of moderated mediation**	0.06	0.03	0.01	0.12

At a low level of community trust, the 95% confidence interval (LLCI = – 0.02, ULCI = 0.17) contains 0, suggesting that digital leadership's impact on community resilience through knowledge sharing was not statistically significant. In contrast, at a high community trust level, the 95% confidence interval (LLCI = 0.06, ULCI = 0.34) excludes 0, demonstrating a statistically significant positive effect of digital leadership on community resilience via knowledge sharing.

Additionally, the positive and significant moderated mediation index (Index = 0.06, LLCI = 0.01, ULCI = 0.12) indicated that community trust significantly moderated the indirect effect of digital leadership and community resilience through knowledge sharing. Hence, H4 of the study was also supported.

## 5 Discussion

### 5.1 Theoretical contributions

This study contributes to the research on community resilience by introducing digital leadership as a new antecedent variable to address a gap in understanding how leadership enhances community resilience in the digital era. Previous research has primarily focused on the positive impact of risk perception (97), social learning (98), and social capital (99, 100) on community resilience. In contrast, our findings indicate that digital leadership significantly and positively influences community resilience, which is consistent with emerging evidence on technology-driven governance (101, 102), yet extends this perspective to community resilience. This challenges the traditional paradigm of leadership as a hierarchical, authority-based approach (103) and positions digital leadership as a democratizing force in disaster management. By helping communities harness decentralized communication networks, digital leadership redefines resource mobilization during crises (12, 104–106).

The mediation of knowledge sharing, grounded in social exchange theory, offers a mechanistic explanation for how digital leadership enhances community resilience. In contrast to qualitative studies that oversimplify this relationship (55), our research empirically demonstrates that digital leadership positively influences altruistic knowledge sharing (107, 108), which subsequently enhances community resilience. Our finding aligns with disaster sociology frameworks that emphasize shared cognition as a key driver of resilience (109). Specifically, when community leaders promote knowledge sharing behaviors, community members learn more disaster mitigation skills, thereby strengthening community resilience. Practically, our finding implies that community training and education programs should prioritize digital literacy for both leaders and residents to optimize knowledge sharing and enhance community resilience (21).

The moderating role of community trust, theorized via MIP-G, deepens understanding of boundary conditions. Our findings reveal that high community trust amplifies the link between digital leadership and knowledge sharing, resonating with literature on trust as a “lubricant” for community residents' pro-sociality (32). In addition, our moderated mediation model clarifies the pathways through which digital leadership enhances community resilience. By integrating resilience theory with knowledge sharing behavioral processes, our model provides a new perspective to understanding community resilience (110).

### 5.2 Managerial inspirations and policy implications

The theoretical contributions of our study highlight the important role of digital leadership, knowledge sharing, and community trust in enhancing community resilience. Based on these insights, our study proposes the following management insights and policy implications that provide actionable guidance for enhancing community resilience in the digital age.

Community leaders should improve their digital capacity by participating in training. Enhancing digital capacity will enable community leaders to better leverage digital tools to deal with community risks and disasters. Community leaders, such as heads of community committees and secretaries of community Party branch committees, are encouraged to actively participate in training programs to improve their digital skills. Community residents should make greater use of digital technology to participate in knowledge sharing activities. Specifically, community residents can use social media platforms such as TikTok and WeChat for disaster knowledge sharing. Through knowledge-sharing activities within their neighborhoods, communities can use their collective wisdom to mitigate disaster risks. Community organizations such as community residents' committees should take measures to enhance community trust. For example, they can establish social media groups, such as WeChat-based community groups, to promote knowledge exchange among residents. Furthermore, community organizations can build public spaces to bring community residents together, thereby increasing opportunities for communication and reinforcing community trust. During crises, community residents are more likely to trust one another, take action for disaster reduction and enhance community resilience.

For policymakers, government agencies should implement policies that encourage community leaders to enhance their digital literacy and competence. This will promote more scientific and rational decision-making in disaster risk management, enabling communities better resilient in the digital era.

### 5.3 Research limitations and prospects

There are some limitations to this study, but it also offers some directions for further investigation in subsequent research.

First, although the statistical analyses indicated that the effect of CMB was not statistically significant and the findings were reliable, the study was still influenced by the inherent limitations of the questionnaire methodology (86). Therefore, it is recommended that future research should incorporate data from various time points and sources, and validate results using a larger sample size. In particular, the respondents in this study were drawn from less developed regions of China. Future research could expand the scope by collecting data from more developed regions to further validate the research model presented in this paper.

Second, regarding the selection of variables, this study considers knowledge sharing as the sole mediating variable. The influence of digital leadership on community resilience may be mediated by elements like organizational agility, digital transformation, digital engagement, job performance, and innovative work behavior, among other factors (111–113). It would be beneficial for future research to investigate the impact of these factors on the relationship between digital leadership and community resilience.

Third, evaluating the moderated mediation model (e.g., digital leadership, knowledge sharing, community trust, and community resilience) in other cultural contexts would be beneficial to test the model's robustness and more general results.

## 6 Conclusion

This study addresses significant gaps in the understanding of how digital leadership enhances community resilience in the digital era.

This study proposes a moderated mediation model to elucidate the mechanisms through which digital leadership enhances community resilience. The findings demonstrate that digital leadership directly strengthens community resilience while simultaneously operating through knowledge sharing as a partial mediator. Notably, community trust emerges as a significant moderator that amplifies the relationship between digital leadership and knowledge sharing. This research addresses three critical gaps in the existing literature: (1) the lack of integrated frameworks connecting digital leadership with community resilience mechanisms, (2) insufficient examination of knowledge sharing role in building community resilience, and (3) limited understanding of community trust moderating these relationships.

In all, this study advances resilience theory in the digital era by providing a moderated mediation model that integrates the concept of digital leadership, the mediating role of knowledge sharing and the moderating effect of community trust. This not only enriches the understanding of community resilience but also promotes practical applications in community risk and emergency management.

## Data Availability

The raw data supporting the conclusions of this article will be made available by the authors, without undue reservation.
