# Correction: How digital leadership impacts community resilience: a moderated mediation model

**DOI:** 10.3389/fpubh.2025.1671312

**Published:** 2025-08-08

**Authors:** Guanhu Zhao, Fazhen Zhao, Xu Hui, Yanan Wu, Xuemei Zhao

**Affiliations:** ^1^School of Management, Lanzhou University, Lanzhou, China; ^2^Research Center for Emergency Management, Lanzhou University, Lanzhou, China; ^3^Lanzhou University Library, Lanzhou, China; ^4^Evidence-Based Medicine Center, School of Basic Medical Sciences, Lanzhou University, Lanzhou, China; ^5^Centre for Evidence-Based Social Science/Center for Health Technology Assessment, School of Public Health, Lanzhou University, Lanzhou, China; ^6^School of Psychology, Guizhou Normal University, Guiyang, China

**Keywords:** digital leadership, community resilience, knowledge sharing, community trust, moderated mediation model

There was a mistake in Table 2. The average variance extracted, composite reliability and Cronbach's α coefficients as published. Table 2 inadvertently omits 9 rows of data (CR2 – CR10) due to an error in the publication process. The corrected Table 2 appears below.

**Table 2:** The average variance extracted, composite reliability and Cronbach's α coefficients.

**Latent variable**	**Items**	**Loadings**	**AVE**	**Composite reliability**	**Cronbach's α**
Digital leadership	DL1	0.696	0.7354	0.9429	0.941
	DL2	0.858			
	DL3	0.843			
	DL4	0.837			
	DL5	0.952			
	DL6	0.935			
Knowledge sharing	KS1	0.849	0.7663	0.9291	0.928
	KS2	0.897			
	KS3	0.906			
	KS4	0.848			
Community trust	CT1	0.820	0.7386	0.8944	0.894
	CT2	0.875			
	CT3	0.882			
Community resilience	CR1	0.875	0.7243	0.9632	0.964
	CR2	0.864			
	CR3	0.798			
	CR4	0.790			
	CR5	0.835			
	CR6	0.833			
	CR7	0.822			
	CR8	0.836			
	CR9	0.921			
	CR10	0.925			

The original version of this article has been updated.

